# Evaluation of a caries prevention programme for preschool children in Switzerland: is the target group being reached?

**DOI:** 10.1186/s12903-021-01969-3

**Published:** 2021-11-30

**Authors:** Anina Mühlemann, Stefanie von Felten

**Affiliations:** 1 Master Programme in Public Health, University of Zurich, Zurich, Switzerland; 2 Department of Biostatistics, Epidemiology, Biostatistics and Prevention Institute, University of Zurich, Zurich, Switzerland

**Keywords:** Programme evaluation, Prevention, Oral health, Early childhood caries (ECC), Switzerland, Preschool children

## Abstract

**Background:**

With the goal of reducing the prevalence of early childhood caries, the city of Zurich, Switzerland, started a specific prevention programme in 2010. All 2-year-olds are invited to a free dental check-up at a local public dental health service before the first legally mandated yearly dental check-up for school children between 4 and 5 years of age (at kindergarten). However, for the success of this prevention programme, it is of particular importance that children at high risk of caries are reached. The objective of our study was to assess the effectiveness of the prevention programme in (1) reaching the children who needed it the most and (2) improving subsequent oral health.

**Methods:**

This retrospective cohort study included all children born between July 1, 2013 and July 15, 2014 who had lived in Zurich between the ages of 23 and 36 months. Socio-economic data were extracted from official school records, and dental health data from public dental clinic records. Binomial and quasi-binomial generalised linear models were used to identify the socio-economic factors associated with toddler check-up attendance and to assess the associations between attendance and caries experience (dmft $\ge$ 1) as well as degree of treatment (proportion m+f out of dmft) at the kindergarten check-up, adjusting for socio-economic factors.

**Results:**

From a total of 4376 children, 2360 (54%) attended the toddler check-up (mean age 2.4 years) and 3452 (79%) had a dental examination at kindergarten (mean age 5.3 years). Non-Swiss origin of the primary caretaker, presence of older siblings, low amount of savings and allocation to certain public dental clinics were associated with a lower odds of attendance. Factors associated with a higher odds of caries experience were similar to those associated with a lower odds of attendance at the toddler check-up, but additionally included low income. Attendance at the toddler check-up was non-significantly associated with a lower odds of caries experience at kindergarten (adjusted OR 0.84, 95% CI from 0.70 to 1.01), but was significantly associated with a higher degree of treatment at this stage (adjusted OR 2.41, 95% CI from 1.79 to 3.24).

**Conclusions:**

Our study suggests that children with a high caries risk are less likely to attend the toddler check-up. Greater effort should be put into reaching these children.

**Supplementary Information:**

The online version contains supplementary material available at 10.1186/s12903-021-01969-3.

## Background

According to the Global Burden of Diseases study [1], 1.76 billion children newly develop dental caries of the primary dentition each year, such that its incidence ranks 5th among all diseases and ages. Early childhood caries (ECC) is defined as the presence of one or more decayed (non-cavitated or cavitated lesions), missing (due to caries) or filled surfaces in any primary tooth of a child under the age of 6 [2]. In two systematic reviews of worldwide articles published between 1998 and 2018, the prevalence of ECC was 17% in 1-year olds and 36% in 2-year olds [2] or 24% in children younger than 3 years and 57.3% in children 3–6 years old [3]. In addition to being a predictor for caries in the permanent dentition, ECC has many adverse effects on children and their families, as it causes acute and chronic pain, hospitalisations, delays of growth/development and a reduced quality of life [2, 4, 5]. In fact, treating ECC under general anesthesia was reported as the most common day surgery in Canada for children aged 12–59 months and represented 31% of all pediatric day surgeries [6]. In England as well, ECC ranks very high on the list of causes for elective hospital admissions in children [7].

In Switzerland, a massive effort to reduce caries in the population, especially in schoolchildren, was initiated in the 1960s. In the canton of Zurich, this resulted in a decrease in the mean dmft value (sum of decayed, missing and filled permanent teeth, [8]) from 12.50 to 1.31 in 14-year-olds between 1964 and 2009 [9]. Nevertheless, severely decayed teeth remain a problem in many young children, particularly those from a low socio-economic and/or migrant background [10, 11]. In fact, ECC can be used as a marker of social inequality [10]. The treatment of caries in Switzerland is not covered by the country’s basic health insurance, with the exception of hospitalisation for acute swelling and pain. As a consequence, dental treatment is highest on the list of medical treatments neglected for economic reasons [12]. However, the unnecessary delay of treatment in children will often lead to pain and multiple tooth extractions, which can cause problems with tooth alignment later on [13].

Annual dental check-ups are legally mandated for children in Zurich starting at the age of 4–5 years at kindergarten (2 years’ mandatory elementary education), and continuing until the age of 15–16 years (end of compulsory education). The annual check-ups are performed either free of charge at six public dental clinics located throughout the city and operated as part of public school services (offered to children by the city of Zurich), or for a fee in private practices. Allocation to the different public dental clinics depends on the home address and, later on, the school the child attends. All check-ups consist of a visual examination of the oral cavity by a dentist, and the dental findings are recorded.

Before 2010, the dental health of preschool children may have been monitored by physicians, other healthcare workers or dentists, but this was probably not the case for the majority of children. The government of Zurich thus started an oral health promotion programme in 2010. All residents with 2-year-old children receive a letter inviting them to a free appointment for their child at one of the public dental clinics (toddler check-up) together with some informational brochures. This invitation is sent out only once and is written in German. The brochures contain information on risk factors for ECC, advice on good oral hygiene practices and dietary counselling, and recommendations on what to do in the event of a dental accident. The optional check-up includes a visual examination of the oral cavity and an educational conversation regarding oral hygiene and dietary habits. At least one parent or caretaker is present. Dentists are briefed on the information that should be communicated during the check-up but receive no formal training. Fluoride varnish is applied where deemed necessary and patients at high risk of caries (based on clinical findings, oral hygiene and dietary habits) or who have already developed caries are advised to visit a dentist at regular intervals.

Children from low-income families, who live with a single parent, with a higher birth order (more older siblings), with less educated parents or who are from ethnic minorities are at higher risk of caries [14]. These children are also more likely to have a history of missed dental appointments and fewer annual check-ups [14, 15]. It is therefore likely that the free toddler check-up offered by the city of Zurich is missed more often by children with a high caries risk. Thus, the aim of this study was to determine the factors associated with attendance (non-attendance) at the free and voluntary toddler check-up and to assess the effectiveness of the check-up in preventing ECC and reducing treatment delays. A further aim was to evaluate the most important socio-economic risk factors for ECC, to enable specifically tailored preventative measures.

We hypothesized that (1) socio-economic factors known to be associated with a higher risk of cavities (in Switzerland) are associated with a lower probability of attendance at the toddler check-up; that attendance at the toddler check-up is associated with (2) a lower risk of cavities and with (3) a higher degree of treatment at the kindergarten check-up; (4) that a migrant background is a stronger predictive factor than economic status for cavities at kindergarten age.

## Methods

The study was designed as a retrospective cohort study. Clarification of responsibility was obtained from the ethics committee of the canton of Zurich (BASEC-Nr. Req-2019-00559).

### Study population

**Fig. 1:**
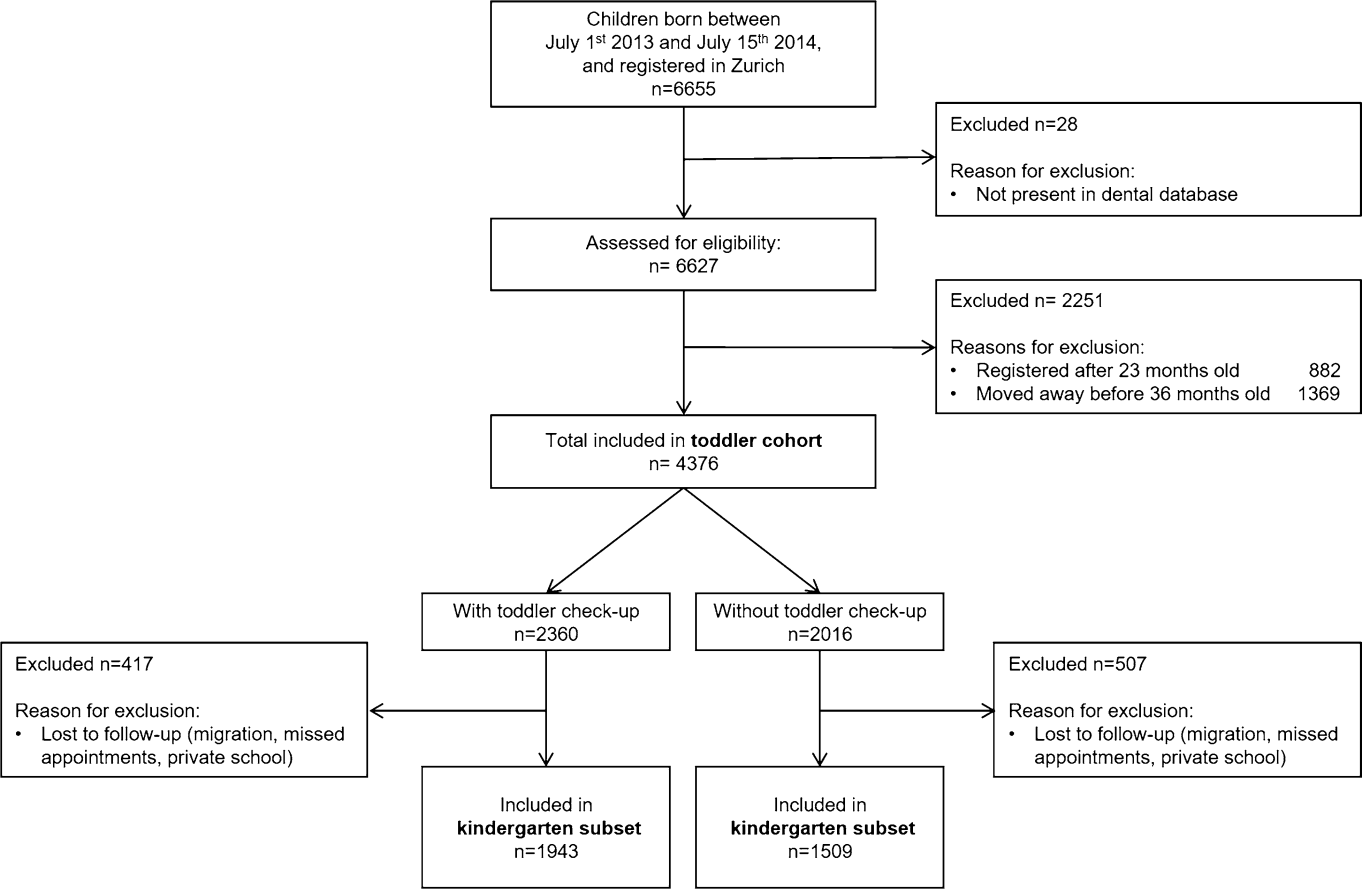
Flow diagram of the study population

The toddler cohort in this study consisted of all children born between July 1, 2013 and July 15, 2014 (children scheduled to start kindergarten in summer 2018) who were registered in the city of Zurich from no later than 23 months of age up to at least 36 months of age. Public dental clinics invited children at slightly different ages: at the earliest shortly before the child’s second birthday and at the latest just before his or her third birthday. The inclusion criterion of registration in Zurich between 23 and 36 months of age ensured that all families had received an invitation for a toddler check-up. The kindergarten subset of the cohort consisted of toddlers for whom data on the later kindergarten check-up (August 2018–December 2019) were available (Fig. 1). We considered the first check-up in this period, which usually occurred during the first year of kindergarten (mostly in spring 2019). However, since children who missed this check-up were allowed to catch up on the missed appointment, we considered check-ups until the middle of second year (December 2019) in case of no earlier check-up. Although the sample size was not formally calculated, we decided that the inclusion of children born within the 1-year span in the toddler cohort would provide enough data to fit the multivariable models planned in advance, following the recommendation of 10 or more events per (explanatory) variable in case of binary outcomes [16].

### Data sources

Public dental clinics in Zurich share a server-based dental software (Vitodent) from which dental findings were obtained, including the dmft index (decayed, missing and filled deciduous teeth). Socio-economic information on the families of the children was obtained from the official Zurich school registry database. An independent data analyst from the public school services extracted and anonymised the data in January 2020.

### Variables

The outcome variables were attendance at the toddler check-up (binary, see hypothesis 1), caries experience at kindergarten age (binary, as dmft $\ge$ 1 vs. dmft = 0, hypotheses 2 and 4) and degree of treatment at kindergarten age (proportion m+f out of dmft, hypothesis 3). The dates of the toddler and kindergarten check-ups were deduced using accounting information (different accounting codes entered for different types of examinations/treatments) from Vitodent. The dmft values were extracted for the toddler and kindergarten check-ups (first check-up during kindergarten). Teeth with clinically detectable carious dentin were entered as decayed. For teeth that were missing, the reason for the absence was recorded (exfoliation, accident or caries). Teeth lost due to normal exfoliation or accidents were not included in the dmft value (not counted as “missing”). As children in need of dental treatment before the kindergarten check-up might also have had X-rays taken, radiologically detectable caries had been added to the dmft value in those cases. For the outcome caries experience at kindergarten, the dmft value at the kindergarten check-up was dichotomised as dmft $\ge$ 1 (vs. dmft = 0) since the dmft value was generally low (dmft = 0 in the majority of children). Degree of treatment was defined as the proportion of missing and filled teeth (m+f out of dmft). Since many upper front teeth were left untreated by choice, the dmft values of those teeth were subtracted from the initial dmft value in the calculation of the degree of treatment.

Explanatory variables for attendance at the toddler check-up were extracted or derived from information in the official Zurich school registry database and included the sex of the child, origin of the primary caretaker, family income, family savings, number of older siblings, living situation of the child (with both parents, mother, father, or neither) and clinic allocation (one of the six public dental clinics). The variable origin of the primary caretaker was created to reflect the cultural background of the primary caretaker, as he or she is largely responsible for the oral health of the child. The origin of the primary caretaker was categorised based on the following characteristics listed in the database: with whom the child lived, the current citizenship of the parents and child and the former citizenship of the parent in case of Swiss naturalisation. The primary caretaker was defined as the mother for children living with their mother or both parents, and the father for children living only with their father. For children who did not live with either parent (e.g., living with other relatives, foster parents or in a children’s home), the child’s origin was recorded. A Swiss origin of the primary caretaker was defined as Swiss citizenship and a Swiss language (German, French, Italian or Romansh) spoken at home (as first language of the child). Swiss caretakers with known migrant background were allocated to their country of origin. Countries of origin of the primary caretaker were then grouped according to assumed caries risk [11], while also aiming to limit the number of groups and thus ensure reasonable group sizes (a list of countries included in each group is provided in Additional file 1). The category “other” mainly included children born to Swiss mothers without known naturalisation and a non-Swiss first language. For the number of older siblings, siblings of the same age (relevant for twins or multiples) were also counted but for simplicity this variable is referred to in the following as the number of older siblings. Taxable income and savings per family were defined as the sum of the incomes and savings of both parents, in case the parents were not married (income, savings and marital status of parents were available in the school registry database). Income was then categorised as < 25,000, 25,000–49,999, 50,000–100,000 and $\ge$ 100,000 Swiss francs (CHF), and savings as < 100,000 vs. $\ge$ 100,000 CHF. Dental treatments at the city of Zurich’s public clinics are not for free, except for children from families registered at the social welfare office, but discounts of 10–50% are provided for taxable incomes < 50,000 CHF, as long as savings are < 100,000 CHF. The cut-offs for the categorisation of income and savings were thus chosen in relation to these discounts. Since data on income and savings were missing for a considerable proportion of the families (completely missing or outdated), two categories, “income missing” and “savings missing” were defined to be able to include the children from those families in the statistical models. Clinic was used as an explanatory variable to assess whether the attendance rates for the toddler check-up differed between public dental clinics even after adjusting for socio-economic variables, either due to characteristics of the clinic or due to unexplained socio-economic variation between catchment areas.

The explanatory variables for caries experience and degree of treatment at kindergarten were attendance at the toddler check-up (exposure of interest) and the same variables as described above to explain attendance at the toddler check-up. Those were considered as confounders, since they were likely related to attendance at the toddler check-up and to the outcomes caries experience and degree of treatment. Age at the kindergarten check-up was used as an additional covariate, because older children would have had more time to develop caries (and get treatment) prior to this check-up.

Additional variables were used to describe the toddler cohort but they were not included in the statistical models due to their similarity with the explanatory variables described above and because they were considered less relevant or less general. These variables included: number of siblings at 2 years old, current citizenship of the primary caretaker, residence permit status of the child and other appointments (dental appointments other than the toddler check-up) until age three.

### Statistical analysis

All statistical analyses were performed using the R system for statistical computing and graphics, version 4.0.4 [17]. The frequencies and percentages of the characteristics of the children in the toddler cohort and their families were tabulated based on attendance at the toddler check-up. A multivariable binary generalised linear model (with binomial error and logit link) was fitted on attendance at the toddler check-up, using the explanatory variables described above.

A similar model was used for the kindergarten subset to analyse the caries experience (dmft $\ge$ 1 vs. dmft = 0) at the kindergarten check-up, with attendance at the toddler check-up and age at the kindergarten check-up as additional explanatory variables. In addition, the degree of treatment at the kindergarten check-up was analysed as the proportion of filled and missing teeth out of dmft for the kindergarten subset, again using a generalised linear model and the same explanatory variables used in the analysis of the caries experience. For this model, a quasi-binomial error distribution (also with logit link) was chosen to account for overdispersion. Unadjusted odds ratios (ORs) were estimated for the association of attendance at the toddler check-up with the outcomes at the kindergarten check-up, to enable comparisons with the adjusted ORs estimated by the multivariable models. All ORs were estimated with a 95% confidence interval (CI).

Finally, the factors that best predicted caries experience at the kindergarten check-up were investigated using a conditional inference tree [18, 19] with all of the previously described explanatory variables for this outcome. Here, income and savings were entered as continuous variables to allow for an estimation of the cut-off values. Each node of the resulting tree represented a two-way split of the (remaining) study population, with the next two subgroups then calculated based on the largest difference in caries prevalence. Further splitting was done as long as the p value was $<0.05$. *P* values were calculated using permutation tests with Bonferroni-adjustment.

Due to the definition of the toddler cohort and the kindergarten subset, there were no children with missing outcome data. Missing values in the explanatory variables income and savings were handled by separate missing categories.

## Results

Among the 6655 children born between July 1, 2013 and July 15, 2014 and registered in the city of Zurich (Fig. 1), 28 were excluded from the study because their data had not been entered in the dental database. A further 2251 children were excluded because they had either registered in the city of Zurich after 23 months of age or they had moved away before 36 months of age. The toddler cohort thus included 4376 children who had been invited to the free dental check-up for toddlers at around 2 years of age. Out of these, 3452 (79%) also belonged to the kindergarten subset, i.e. they had attended a check-up during kindergarten at one of the public dental clinics. The mean age of the children was 2.4 years (standard deviation, SD, 0.4) at the toddler check-up and 5.3 years (SD 0.3) at the kindergarten check-up.

### Attendance at the toddler check-up

In the toddler cohort, 2360 of the 4376 children (54%) had attended the toddler check-up at a public dental clinic while 2016 children had not (Fig. 1). Children who had attended the toddler check-up differed considerably from those who had not regarding all characteristics except sex (Table 1). In the multivariate model (adjusting each variable by all others), several factors were associated with a lower odds of attendance at the toddler check-up: primary caretaker of non-Swiss origin (OR for Eastern Europe/Turkey/Russia < Western Europe/Scandinavia/USA/CAN/AUS/NZL, collectively referred to hereafter as “Western” < South America/Africa/Asia) as opposed to Swiss origin, the number of older siblings (OR decreased with increasing number of siblings), living with neither parent, and allocation to certain public dental clinics (Table 2). By contrast, savings $\ge$ 100,000 CHF were associated with a higher odds of attendance (Table 2). A descriptive comparison of the socio-economic background of the clinics’ clientele is shown in Additional file 2.

**Table 1:** Characteristics of the children in the toddler cohort and their families by attendance at the toddler check-up

Variable	Overall	No toddler check-up	Toddler check-up	Missing (%)
n	4376	2016	2360	
Female (%)	2172 (49.6)	1007 (50.0)	1165 (49.4)	0.0
Origin of primary caretaker (%)				0.0
Switzerland	1924 (44.0)	750 (37.2)	1174 (49.7)	
Western$^{\rm a}$	943 (21.5)	423 (21.0)	520 (22.0)	
South America, Africa, Asia	627 (14.3)	335 (16.6)	292 (12.4)	
Eastern Europe, Turkey, Russia	666 (15.2)	393 (19.5)	273 (11.6)	
Other$^{\rm b}$	216 (4.9)	115 (5.7)	101 (4.3)	
Income (%)				0.0
< 25’000	755 (17.3)	394 (19.5)	361 (15.3)	
25’000-49’999	793 (18.1)	396 (19.6)	397 (16.8)	
50’000-99’999	1363 (31.1)	597 (29.6)	766 (32.5)	
$\ge$ 100’000	1213 (27.7)	491 (24.4)	722 (30.6)	
Income missing	252 (5.8)	138 (6.8)	114 (4.8)	
Savings (%)				0.0
< 100’000	2123 (48.5)	1114 (55.3)	1009 (42.8)	
$\ge$ 100’000	1848 (42.2)	680 (33.7)	1168 (49.5)	
Savings missing	405 (9.3)	222 (11.0)	183 (7.8)	
Number of siblings older/same age (%)				0.0
0	2182 (49.9)	927 (46.0)	1255 (53.2)	
1	1577 (36.0)	729 (36.2)	848 (35.9)	
2	438 (10.0)	241 (12.0)	197 (8.3)	
$\ge$ 3	179 (4.1)	119 (5.9)	60 (2.5)	
Number of siblings when 2 years old (%)				0.0
0	1827 (41.8)	763 (37.8)	1064 (45.1)	
1	1834 (41.9)	845 (41.9)	989 (41.9)	
2	511 (11.7)	274 (13.6)	237 (10.0)	
$\ge$ 3	204 (4.7)	134 (6.6)	70 (3.0)	
Living situation (%)				0.0
Mother and father	3716 (84.9)	1684 (83.5)	2032 (86.1)	
Mother	565 (12.9)	277 (13.7)	288 (12.2)	
Father	45 (1.0)	21 (1.0)	24 (1.0)	
Neither parent	50 (1.1)	34 (1.7)	16 (0.7)	
Clinic allocation (%)				0.0
SAU	747 (17.1)	263 (13.0)	484 (20.5)	
SUS	708 (16.2)	244 (12.1)	464 (19.7)	
SPA	625 (14.3)	265 (13.1)	360 (15.3)	
SWE	540 (12.3)	254 (12.6)	286 (12.1)	
SMU	527 (12.0)	231 (11.5)	296 (12.5)	
SNO	1229 (28.1)	759 (37.6)	470 (19.9)	
Current citizenship of primary caretaker (%)				0.0
Switzerland	2727 (62.3)	1161 (57.6)	1566 (66.4)	
Western	745 (17.0)	345 (17.1)	400 (16.9)	
South America, Africa, Asia	452 (10.3)	241 (12.0)	211 (8.9)	
Eastern Europe, Turkey, Russia	362 (8.3)	218 (10.8)	144 (6.1)	
Other	90 (2.1)	51 (2.5)	39 (1.7)	
Residence permit status of child (%)				0.5
Swiss resident	3260 (74.5)	1430 (70.9)	1830 (77.5)	
Foreign resident	792 (18.1)	416 (20.6)	376 (15.9)	
Foreign resident weekly/yearly permit	260 (5.9)	133 (6.6)	127 (5.4)	
Foreign resident short stay	11 (0.3)	5 (0.2)	6 (0.3)	
Asylum seeker, temporary asylum granted	32 (0.7)	19 (0.9)	13 (0.6)	
Missing	21 (0.5)	13 (0.6)	8 (0.3)	
Other appointments until 3 years old (%)	1470 (33.6)	301 (14.9)	1169 (49.5)	0.0

$^{\rm a}$ Western includes the countries in Western Europe (except Switzerland), Scandinavia, USA, CAN, AUS and NZL.

$^{\rm b}$ Mainly Swiss mothers without known naturalization, but other language spoken at home

### Caries experience at kindergarten

At the kindergarten check-up, 752 of 3452 children (22%) had caries experience (dmft $\ge$ 1), including 345 of 1943 (18%) who had attended the toddler check-up and 407 of 1509 (27%) who had not. The unadjusted OR for caries experience at the kindergarten check-up among children who had attended the toddler check-up vs. those who had not was 0.58 (95% CI, from 0.50 to 0.69), indicating a potential benefit of the toddler check-up. However, this association was weaker and not statistically significant in the multivariable model (adjusted OR 0.84, 95% CI from 0.70 to 1.01, Table 3).

The socio-economic factors associated with a higher odds of caries experience were similar to those associated with a lower odds of attendance at the toddler check-up (see Additional file 3 for descriptive statistics for children with/without caries at the kindergarten check-up). Specifically, a non-Swiss origin of the primary caretaker, one or more older siblings and age at the kindergarten check-up were associated with a higher odds of caries experience, whereas higher income and savings and allocation to the SMU clinic (located in an affluent neighbourhood) were associated with a lower odds of caries experience (Table 3).

**Table 2:** Odds ratio (OR) estimates for associations of socioeconomic characteristics with attendance at the toddler check-up in the toddler cohort

	OR	95% CI	p value
Sex: Male	1	–	–
Sex: Female	0.98	From 0.87 to 1.11	0.81
Origin of primary caretaker: Switzerland	1	–	–
‘Origin of primary caretaker‘Western	0.78	From 0.66 to 0.92	0.004
‘Origin of primary caretaker‘South America, Africa, Asia	0.79	From 0.65 to 0.97	0.026
‘Origin of primary caretaker‘Eastern Europe, Turkey, Russia	0.63	From 0.52 to 0.77	< 0.0001
‘Origin of primary caretaker‘Other	0.84	From 0.62 to 1.14	0.27
Income: < 25’000	1	–	–
Income25’000-49’999	1.02	From 0.83 to 1.26	0.85
Income50’000-99’999	1.09	From 0.89 to 1.33	0.42
Income$\ge$ 100’000	1.01	From 0.81 to 1.27	0.90
IncomeIncome missing	1.42	From 0.90 to 2.25	0.14
Savings: < 100’000	1	–	–
Savings$\ge$ 100’000	1.57	From 1.35 to 1.84	< 0.0001
SavingsSavings missing	0.85	From 0.58 to 1.23	0.38
Number of siblings older/same age: 0	1	–	–
‘Number of siblings older/same age‘1	0.87	From 0.76 to 1.00	0.043
‘Number of siblings older/same age‘2	0.69	From 0.56 to 0.86	0.0008
‘Number of siblings older/same age‘$\ge$ 3	0.38	From 0.27 to 0.53	< 0.0001
Living situation: Mother and father	1	–	–
‘Living situation‘Mother	0.96	From 0.77 to 1.19	0.72
‘Living situation‘Father	1.03	From 0.55 to 1.92	0.94
‘Living situation‘Neither parent	0.32	From 0.15 to 0.63	0.001
Clinic allocation: SAU	1	–	–
‘Clinic allocation‘SUS	0.88	From 0.71 to 1.10	0.26
‘Clinic allocation‘SPA	0.73	From 0.58 to 0.91	0.006
‘Clinic allocation‘SWE	0.63	From 0.50 to 0.79	< 0.0001
‘Clinic allocation‘SMU	0.58	From 0.46 to 0.73	< 0.0001
‘Clinic allocation‘SNO	0.36	From 0.30 to 0.44	< 0.0001

*P* values were derived by Wald z-tests. The model was based on 4376 children (2360 with a toddler check-up)

**Fig. 2:**
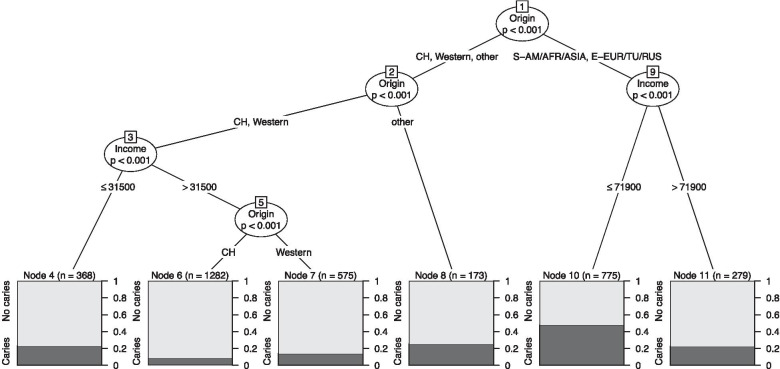
Conditional inference tree to identify the factors most strongly associated with caries experience by the time of kindergarten check-up in the “kindergarten subset”. The label Western includes the countries in Western Europe (except Switzerland), Scandinavia, USA, CAN, AUS and NZL. S-AM/AFR/ASIA stands for South America/Africa/Asia and E-EUR/TU/RUS for Eastern Europe/Turkey/Russia. *P* values were calculated using permutation tests with Bonferroni-adjustment. A total of 3452 children (752 with caries) were included in the tree. The bar charts at the bottom show the caries prevalence for the groups of children defined by the nodes of the tree

The conditional regression tree on caries experience at kindergarten is shown in Fig. 2. A migrant background was the factor most strongly associated with caries experience at the kindergarten check-up, as it defined the first node of the tree. Children with a primary caretaker of Eastern European/Turkish/Russian or Asian/African/South American origin (right branch) had a higher odds of caries experience than children whose primary caretaker was Swiss, Western or other origin (left branch), especially if the family income was $\le$ 71,900 CHF. The left branch was then further split depending on origin and income. The bar charts at the bottom of the tree show the caries prevalence for the groups of children defined by the nodes of the tree. Caries prevalence was lowest in children whose primary caretaker was of Swiss origin and whose family had an income > 31,000 CHF.

### Degree of treatment at kindergarten

Degree of treatment at the kindergarten check-up was on average 0.52 in children with caries who had attended the toddler check-up and 0.29 in children with caries who had not. Both the unadjusted and adjusted OR for degree of treatment at the kindergarten check-up among children who had attended the toddler check-up vs. those who had not indicate a benefit of the toddler check-up (unadjusted OR 2.49, 95% CI from 1.90 to 3.27, adjusted OR 2.41, 95% CI from 1.79 to 3.24, Table 4). Moreover, non-Swiss origin of the primary caretaker (OR for South America/Africa/Asia < Eastern Europe/Turkey/Russia < Western) was negatively associated but age at the kindergarten check-up was positively associated with the degree of treatment. There was no evidence of an association between income/savings and the degree of treatment.

**Table 3:** Odds ratio (OR) estimates for associations between caries experience (dmft $\ge$ 1 vs. dmft = 0) and socioeconomic characteristics, attendance at the toddler check-up and age at kindergarten check-up in the kindergarten subset

	OR	95% CI	p value
Sex: Male	1	–	–
Sex: Female	0.94	from 0.79 to 1.12	0.47
Origin of primary caretaker: Switzerland	1	–	–
‘Origin of primary caretaker‘Western	1.61	From 1.22 to 2.10	0.0006
‘Origin of primary caretaker‘South America, Africa, Asia	3.57	From 2.75 to 4.65	< 0.0001
‘Origin of primary caretaker‘Eastern Europe, Turkey, Russia	4.67	From 3.62 to 6.04	< 0.0001
‘Origin of primary caretaker‘Other	2.28	from 1.52 to 3.39	< 0.0001
Income: < 25’000	1	–	–
Income25’000-49’999	0.76	From 0.58 to 0.98	0.038
Income50’000-99’999	0.60	From 0.46 to 0.77	< 0.0001
Income $\ge$ 100’000	0.44	From 0.31 to 0.61	< 0.0001
IncomeIncome missing	0.90	From 0.48 to 1.70	0.74
Savings: < 100’000	1	–	–
Savings$\ge$ 100’000	0.63	From 0.50 to 0.80	0.0002
SavingsSavings missing	0.78	From 0.45 to 1.32	0.36
Number of siblings older/same age: 0	1	–	–
‘Number of siblings older/same age‘1	1.30	From 1.07 to 1.58	0.009
‘Number of siblings older/same age‘2	1.47	From 1.10 to 1.95	0.009
‘Number of siblings older/same age‘$\ge$ 3	2.24	From 1.53 to 3.28	< 0.0001
Living situation: Mother and father	1	–	–
‘Living situation‘Mother	1.02	From 0.76 to 1.37	0.88
‘Living situation‘Father	0.96	From 0.36 to 2.28	0.94
‘Living situation‘Neither parent	2.68	from 0.62 to 10.67	0.16
Clinic allocation: SAU	1	–	–
‘Clinic allocation‘SUS	0.91	From 0.65 to 1.27	0.58
‘Clinic allocation‘SPA	1.06	From 0.78 to 1.45	0.70
‘Clinic allocation‘SWE	1.14	From 0.83 to 1.56	0.43
‘Clinic allocation‘SMU	0.57	From 0.37 to 0.87	0.01
‘Clinic allocation‘SNO	0.97	From 0.74 to 1.28	0.83
‘Age at kindergarten check-up‘	1.54	From 1.18 to 2.02	0.002
‘Toddler check-up‘	0.84	From 0.70 to 1.01	0.071

*P* values were derived by Wald z-tests. The model was based on 3452 children (752 with caries)

### Caries prevalence

Caries prevalence at the toddler check-up (in children from the toddler cohort who attended) was 3.4% (95% CI from 2.7 to 4.2%), with a mean dmft value of 0.13 (range 0–13). Among the individual components of the dmft index in children with caries, the percentages were as follows: d = 93%, m = 6% and f = 1%. Caries prevalence at kindergarten (kindergarten subset) was 21.8% (95 % CI from 20.4 to 23.2%), with a mean dmft value of 0.89 (range 0–18) and d = 64%, m = 6%, f = 30%. Caries prevalence at kindergarten for the subgroups of children classified by the conditional regression tree is shown in Fig. 2. The distributions of dmft values at the toddler and kindergarten check-ups are shown in Additional file 4.

## Discussion

This study provides strong evidence for an association between socio-economic variables and attendance at the toddler check-up. Moreover, our hypothesis (1) that socio-economic factors known to be associated with a higher risk of cavities (in Switzerland) are associated with a lower probability of attendance at the toddler check-up, was confirmed; factors associated with a lower odds of attendance were similar to those associated with a higher odds of caries experience at kindergarten (Tables 2 and 3). Children from primary caretakers of Eastern European/Turkish/Russian, Western, or South American/African/Asian origin were less likely to attend than those with primary caretakers of Swiss origin. This also applied to children from families with less savings and children with two or more older siblings. Children allocated to certain public dental clinics (thus living in certain districts in Zurich) also had lower attendance rates. Our hypothesis (2) that attendance at the toddler check-up is associated with a lower risk of cavities was only partially confirmed, since the odds for caries experience at the kindergarten check-up was lower in those who attended at the toddler check-up (adjusted for socio-economic factors), but the difference was non-significant (Table 3). Our hypothesis (3) that attendance at the toddler check-up is associated with a higher degree of treatment at the kindergarten check-up was confirmed, since degree of treatment was higher in those who attended at the toddler check-up (also adjusted for for socio-economic factors, Table 4). Finally, our hypothesis (4) that a migrant background is a stronger predictive factor than economic status for cavities at kindergarten age was confirmed; the origin of the primary caretaker played the most important role in the child’s early development of caries, followed by family income (Fig. 2).

**Table 4:** Odds ratio (OR) estimates for associations between the degree of treatment (proportion of filled or missing teeth out of dmft) and socioeconomic characteristics, attendance at the toddler check-up and age at kindergarten check-up in the kindergarten subset

	OR	95% CI	p value
Sex: Male	1		
Sex: Female	1.10	from 0.82 to 1.46	0.54
Origin of primary caretaker: Switzerland	1		
‘Origin of primary caretaker‘Western	0.44	From 0.25 to 0.80	0.007
‘Origin of primary caretaker‘South America, Africa, Asia	0.35	From 0.22 to 0.56	< 0.0001
‘Origin of primary caretaker‘Eastern Europe, Turkey, Russia	0.43	From 0.27 to 0.67	0.0003
‘Origin of primary caretaker‘Other	0.51	From 0.24 to 1.05	0.068
Income: < 25’000	1		
Income25’000-49’999	0.92	From 0.62 to 1.38	0.70
Income50’000-99’999	0.94	From 0.62 to 1.42	0.76
Income$\ge$ 100’000	1.38	From 0.73 to 2.61	0.33
IncomeIncome missing	1.72	From 0.64 to 4.66	0.28
Savings: < 100’000	1		
Savings$\ge$ 100’000	0.93	From 0.58 to 1.49	0.77
SavingsSavings missing	0.93	From 0.37 to 2.28	0.87
Number of siblings older/same age: 0	1		
‘Number of siblings older/same age‘1	0.99	From 0.71 to 1.39	0.96
‘Number of siblings older/same age‘2	0.89	From 0.58 to 1.38	0.61
‘Number of siblings older/same age‘$\ge$ 3	1.20	From 0.71 to 2.02	0.49
Living situation: Mother and father	1		
‘Living situation‘Mother	0.98	From 0.62 to 1.56	0.94
‘Living situation‘Father	3.03	From 0.57 to 21.30	0.21
‘Living situation‘Neither parent$^{\rm a}$	–	–	–
Clinic allocation: SAU	1		
‘Clinic allocation‘SUS	1.45	From 0.79 to 2.66	0.24
‘Clinic allocation‘SPA	1.42	From 0.85 to 2.39	0.19
‘Clinic allocation‘SWE	1.03	From 0.63 to 1.67	0.92
‘Clinic allocation‘SMU	1.37	From 0.58 to 3.21	0.46
‘Clinic allocation‘SNO	1.23	From 0.80 to 1.90	0.35
‘Age at kindergarten check-up‘	3.92	From 2.43 to 6.40	< 0.0001
‘Toddler check-up‘	2.41	From 1.79 to 3.24	< 0.0001

Note that upper front teeth were excluded since they are often left untreated. *P* values were derived by Student t-tests. The model was based on 3452 children (329 with filled or missing teeth). The proportion of filled or missing teeth was between 0 and 1.00

$^{\rm a}$ OR could not be estimated due to the very small number of children in this group (perfect separation problem)

Our findings on caries experience are in line with those reported in a systematic review by Hooley et al. [14], in which 84% of the studies found that the sociodemographic factors of families were significantly associated with ECC. The identified risk factors included lower socio-economic class, lower family income, immigrant status, parents with a low level of education and unemployed parents. Many studies also reported an association between the number of siblings in the family, especially being a sibling of higher birth order, and unfavourable health outcomes of the children, including a higher risk of caries experience and a lower level of medical surveillance and immunisation [14]. A role of siblings and birth order in caries experience and attendance at the toddler check-up was also determined in our study. The possible reasons for this finding include parents’ limited amount of time and energy and thus a neglect of the oral health of their children, limited financial resources, differences in cultural beliefs, but also simply the fact that the parents believe to know enough about caries and oral health. Our results also support the finding of Hooley et al. [14] that children with caries are less likely to regularly visit a dentist. Moreover, they are in line with the *inverse equity hypothesis* [20], postulating that “new public-health interventions and programmes initially reach those of higher socio-economic status and only later affect the poor”.

Our finding of only a slight trend for a preventive effect of the toddler check-up on caries experience 2–4 years later is in contrast to the results of Wagner et al. [21, 22] and Winter et al. [23], both of whom reported a significant preventive effect of one intervention as well as recurring interventions on the risk of caries experience in children in Germany and Austria. A possible explanation is that the onset of these programmes was just after birth and that they included the continuous and systematic training of participating health workers and dentists. In addition, our cohort included several children who were already severely affected by ECC at the toddler check-up (Additional file 4). Overall, these findings support the recommendations of systematic reviews, that interventions aimed at preventing caries should occur during the first 2 years of a child’s life [24] and should start no later than age 12 months [2]. The strategies of the British Society of Paediatric Dentistry (BSPD), the American Academy of Pediatric Dentistry and the Canadian Dental Association, who all encourage to have a first dental check by 12 months of age [25–27] point in the same direction. In contrast, the Swiss Dental Association recommends a first dental check for children at the age of 2 years [28]. The importance of an early dental check for children and the challenges in implementing this strategy in the United Kingdom were emphasized in a recent editorial by Ivor G. Chestnutt entitled “How soon is soon enough?” [29].

Contrary to the results of Wolff et al. [12] we found no evidence for an association between the degree of treatment and either income or savings, perhaps due to the financial aid for dental treatment given to low-income families in Zurich. In addition, the small number of children with dmft $\ge$ 1 in the highest income and savings groups may have hindered the detection of statistically significant differences regarding the degree of treatment.

The prevalence of caries at the toddler check-up (3.4%) was considerably lower than the 12.6% reported by Menghini et al. [11], who examined 771 out of a random sample of 1000 children in Zurich in the year 2003 (mean age 2.4 years). Moreover, the association between attendance at the toddler check-up and caries experience at the kindergarten check-up became noticeably weaker after adjusting for caries risk factors. Together, these findings suggest a selection bias, as children with a low caries risk may have been more likely to attend the toddler check-up than children with a high caries risk, in line with the results of Hooley et al. [14]. Other possible explanations for the low caries prevalence in our study are failed attempts to clinically examine the small children at the toddler check-up, resulting in an overestimation of children with dmft = 0, and the success of more recent caries prevention programmes. The latter is supported by our findings from the kindergarten check-up, in which the mean dmft value was 0.89 and 21.8% of children had a dmft $\ge$ 1. By contrast, the mean dmft value for 5-year-olds in Zurich (age 4.5–5.5 years) in 2015 was 1.3, when 26% of the children had a dmft $\ge$ 1 [30]. Compared to our results from the kindergarten check-up, and thus slightly older children (mean age 5.30, interquartile range: 5.03–5.57), a small decrease in caries prevalence may be postulated.

The strong association between attendance at the toddler check-up and clinic allocation when adjusted for all other variables (Table 2) may point to differences among the clinics (e.g. the way the check-ups are done, the reputation of the clinic) or alternatively, to differences among the clinics catchment areas and thus failure of our study to adjust for all relevant socio-economic variables. Indeed, clinic allocation may harbour socio-economic information in addition to the information we included. For example, the parents’ educational level was not available in our study, but may have been relevant for participation in the toddler check-up.

The results of our study raise the question of how children in Zurich with a high caries risk can be better reached. Financial barriers are unlikely to limit participation, given that the toddler check-up is free, although poor families may be unable to afford the price of public transportation needed to reach the clinic, if it is not in walking distance. Also, since the invitation letter is written in German, readers with limited understanding of German may not understand the purpose of the check-up and that it is free. An expectation of language barriers in the conversation with the dentist may also prevent attendance. Further, working parents may be unable to take time off from work to bring the child to the check-up. Lastly, some parents may understand the invitation but do not recognise the importance of caries prevention in their children. So far, there are no data on reasons why families may not attend the free toddler check-up with their child in Zurich. We thus recommend a qualitative study, similar to the one conducted for the “Free First Visit” programme in Manitoba, Canada [31]. Moreover, efforts should be made to raise awareness of the necessity of caries prevention and to alert parents to the benefits of the free toddler check-up when their children are still infants, particularly for parents with children at a high risk of caries.

This study focussed on toddlers and kindergarten-age children in the city of Zurich. Because of cultural differences and differences in the characteristics of the population among the different parts of Switzerland, our results might not be generalisable to the whole country. Furthermore, access to dental health care may vary between different regions; indeed, only some Swiss cities have public dental clinics, while in other regions general dentists are responsible for treating children. Finally, the existence and amount of financial aid for dental treatments differ in each community, which could strongly influence the degree of treatment.

While the large cohort of children, the availability of relatively detailed socio-economic information on their families and the possibility to link this information with the public dental clinic records are strengths of this study, it also has several limitations. Most if not all of them were due to the retrospective cohort design of the study. First, the dmft index at the toddler check-up was known only for the children who attended the toddler check-up. As a consequence, dmft index at the toddler check-up could not be included as baseline in the statistical models assessing the association between attendance at the toddler check-up and caries experience or degree of treatment at kindergarten. Since the dmft index at the toddler check-up may have affected both, attendance at the toddler check-up and dmft index at kindergarten, a definitive conclusion regarding the effectiveness of the toddler check-up was not possible. Within the cohort of studied children, our analyses of caries experience and degree of treatment at kindergarten were basically cross-sectional, with attendance at the toddler check-up being the only variable determined at an earlier time. In addition, information on other relevant covariates, such as the educational level, employment status, dental health awareness and motivation of the parents, was missing. Thus, some degree of residual confounding in estimates of the adjusted ORs for attendance at the toddler check-up on outcomes at the kindergarten check-up is likely. Second, data on the income and savings of the parents were sometimes outdated and incomplete, leading to missing values for both. Although the use of separate “missing categories” allowed us to include the children of those families in our statistical models, the groups with missing income or savings are themselves ambiguous. Third, the outcomes caries experience and degree of treatment were based on the dmft index and may therefore have been imprecise, since the number of cavities may be underestimated when based on visual examination alone. This would have been more relevant in children without previous dental treatment and therefore no X-ray images of the oral cavity. As this was probably more common in children of higher socio-economic status, an underestimation of caries was more likely in this group, and the overall benefit of the prevention programme may therefore have been overestimated. Moreover, as there was no standardised training of the dentists regarding the documentation of caries levels, it was possible that non-cavitated carious teeth were not documented consistently. Overall, the quality of both the visual examination and the documentation of the results depended on the dentist and on patient cooperation. Finally, loss to follow-up between the toddler cohort and the kindergarten subset was high (21%: 924 out of 4376 children), leading to a risk of selection bias.

## Conclusions

An at least partial effectiveness of the optional dental check-up for toddlers in Zurich, Switzerland, can be inferred, due to the association of attendance with a higher degree of treatment and a tendency for a lower odds of caries experience at kindergarten. However, our study clearly indicates that children with a high caries risk are less likely to attend the toddler check-up. Greater effort should therefore be made to reach these children. In addition, an earlier onset of the toddler check-up may improve subsequent dental health.

## Supplementary Information

**Additional file 1.** List of countries of origin allocated to each origin of primary caretaker group.

**Additional file 2.** Characteristics of families of children in the toddler cohort by public dental clinic allocation.

**Additional file 3.** Characteristics of children and their families in the kindergarten subset by presence of caries atthe kindergarten check-up.

**Additional file 4.** Frequency distribution of dmft indices at the toddler and kindergarten check-ups.

## Data Availability

The datasets collected and analysed during the current study are not publicly available, since the authors do not own the data and had to sign a confidentiality statement preventing them from giving the data to third parties. The data may be requested from the head of public dental services of the city of Zurich, Dr. Hubertus van Waes (hubertus.vanwaes@zuerich.ch).

## Abbreviations

CHF: Swiss franc
CI: Confidence interval
DMFT index: Decayed, missing, or filled permanent teeth
dmft index: Decayed, missing, or filled deciduous teeth
ECC: Early childhood caries
OR: Odds ratio
SD: Standard deviation
