# Supplementary material for: Evaluation of a caries prevention programme for preschool children in Switzerland: is the target group being reached?
Source: BMC Oral Health. 2021 Nov 30;21:609. doi: 10.1186/s12903-021-01969-3 (PMC8638191; doi:10.1186/s12903-021-01969-3)
Supplement: Supplementary file 1 — Additional file 1. List of countries of origin allocated to each origin of primary caretaker group. [file 12903_2021_1969_MOESM1_ESM.pdf]

Evaluation of a caries prevention programme for preschool children in Switzerland: Is the target group being reached?

Countries of origin of the primary caretaker

Countries are grouped as in our study and are listed in alphabetic order

Swiss: Switzerland

Western-Europe, Scandinavia, USA/CAN, AUS, NZL: Australia, Austria, Belgium, Canada, Denmark, France, Finland, Germany, Italy, Ireland, Iceland, Liechtenstein, Luxembourg, Netherlands, New Zealand, Norway, Portugal, Spain, Sweden, UK, USA

Eastern-Europe, Turkey, Russia: Albania, Belarus, Bosnia-Herzegovina, Bulgaria, Croatia, CSFR (Czech and Slovak Federative Republic), Cyprus, Czech Republic, Estonia, Greece, Georgia, Hungary, Kosovo, Latvia, Lithuania, Macedonia, Malta, Moldova, Montenegro, Poland, Romania, Russia, Serbia, Serbia and Montenegro, Slovak Republic, Slovenia, Turkey, Ukraine, Yugoslavia

South America, Africa, Asia: Afghanistan, Algeria, Angola, Argentina, Armenia, Azerbaijan, Bangladesh, Bolivia, Brazil, Burkina Faso, Cambodia, Cameroon, Chile, China, Colombia, Congo, Costa Rica, Cote d'Ivoire, Cuba, Democratic Republic of Congo, Dominican Republic, Ecuador, Egypt, El Salvador, Eritrea, Ethiopia, Gambia, Ghana, Guatemala, Guinea, Guinea Bissau, Honduras, India, Indonesia, Iran, Iraq, Israel, Jamaica, Japan, Jordan, Kazakhstan, Kenya, Kyrgyzstan, Laos, Lebanon, Libya, Malaysia, Mali, Mauritius, Mexico, Mongolia, Morocco, Mozambique, Nepal, Nigeria, Pakistan, Palestine, Panama, Paraguay, Peru, Philippines, Republic of Korea, Rwanda, Saudi Arabia, Senegal, Singapore, Somalia, South Africa, Sri Lanka, St. Kitts and Nevis, Sudan, Syria, Thailand, Taiwan, Tanzania, Trinidad and Tobago, Tunisia, Uruguay, Uzbekistan, Venezuela, Vietnam, Zambia, Zimbabwe
