# Supplementary material for: Evaluation of a caries prevention programme for preschool children in Switzerland: is the target group being reached?
Source: BMC Oral Health. 2021 Nov 30;21:609. doi: 10.1186/s12903-021-01969-3 (PMC8638191; doi:10.1186/s12903-021-01969-3)
Supplement: Supplementary file 2 — Additional file 2. Characteristics of families of children in the toddler cohort by public dental clinic allocation. [file 12903_2021_1969_MOESM2_ESM.pdf]

Evaluation of a caries prevention programme for preschool children in Switzerland: Is the target group being reached?

Table 1: Characteristics of families of children in the toddler cohort by public dental clinic allocation.

**Variable**	**SAU**	**SUS**	**SPA**	**SWE**	**SMU**	**SNO**
n	747	708	625	540	527	1229
Origin of primary caretaker (%)						
Switzerland	360 (48.2)	403 (56.9)	314 (50.2)	207 (38.3)	278 (52.8)	362 (29.5)
Western	164 (22.0)	175 (24.7)	143 (22.9)	100 (18.5)	158 (30.0)	203 (16.5)
South America, Africa, Asia	108 (14.5)	50 (7.1)	83 (13.3)	94 (17.4)	38 (7.2)	254 (20.7)
Eastern Europe, Turkey, Russia	84 (11.2)	53 (7.5)	65 (10.4)	109 (20.2)	37 (7.0)	318 (25.9)
Other	31 (4.1)	27 (3.8)	20 (3.2)	30 (5.6)	16 (3.0)	92 (7.5)
Income (%)						
<25’000	165 (22.1)	81 (11.4)	121 (19.4)	116 (21.5)	46 (8.7)	226 (18.4)
25’000-49’999	158 (21.2)	108 (15.3)	105 (16.8)	106 (19.6)	50 (9.5)	266 (21.6)
50’000-99’999	204 (27.3)	241 (34.0)	188 (30.1)	191 (35.4)	149 (28.3)	390 (31.7)
≥100’000	168 (22.5)	250 (35.3)	181 (29.0)	98 (18.1)	265 (50.3)	251 (20.4)
Income missing	52 (7.0)	28 (4.0)	30 (4.8)	29 (5.4)	17 (3.2)	96 (7.8)
Savings (%)						
<100’000	370 (49.5)	275 (38.8)	298 (47.7)	308 (57.0)	156 (29.6)	716 (58.3)
≥100’000	290 (38.8)	388 (54.8)	279 (44.6)	184 (34.1)	337 (63.9)	370 (30.1)
Savings missing	87 (11.6)	45 (6.4)	48 (7.7)	48 (8.9)	34 (6.5)	143 (11.6)
