# Supplementary material for: Evaluation of a caries prevention programme for preschool children in Switzerland: is the target group being reached?
Source: BMC Oral Health. 2021 Nov 30;21:609. doi: 10.1186/s12903-021-01969-3 (PMC8638191; doi:10.1186/s12903-021-01969-3)
Supplement: Supplementary file 3 — Additional file 3. Characteristics of children and their families in the kindergarten subset by presence of caries atthe kindergarten check-up. [file 12903_2021_1969_MOESM3_ESM.pdf]

Evaluation of a caries prevention programme for preschool children in Switzerland: Is the target group being reached?

Table 1: Characteristics of children and their families in the kindergarten subset by caries experience at the kindergarten check-up.

**Variable**	**Overall**	**No caries**	**Caries**	**Missing (%)**
n	3452	2700	752	
Female (%)	1749 (50.7)	1380 (51.1)	369 (49.1)	0.0
Origin of primary caretaker (%)				0.0
Switzerland	1553 (45.0)	1383 (51.2)	170 (22.6)	
Western	672 (19.5)	567 (21.0)	105 (14.0)	
South America, Africa, Asia	538 (15.6)	325 (12.0)	213 (28.3)	
Eastern Europe, Turkey, Russia	516 (14.9)	296 (11.0)	220 (29.3)	
Other	173 (5.0)	129 (4.8)	44 (5.9)	
Income (%)				0.0
<25’000	610 (17.7)	389 (14.4)	221 (29.4)	
25’000-49’999	658 (19.1)	483 (17.9)	175 (23.3)	
50’000-99’999	1139 (33.0)	931 (34.5)	208 (27.7)	
≥100’000	896 (26.0)	803 (29.7)	93 (12.4)	
Income missing	149 (4.3)	94 (3.5)	55 (7.3)	
Savings (%)				0.0
<100’000	1759 (51.0)	1247 (46.2)	512 (68.1)	
≥100’000	1431 (41.5)	1273 (47.1)	158 (21.0)	
Savings missing	262 (7.6)	180 (6.7)	82 (10.9)	
Number of siblings older/same age (%)				0.0
0	1616 (46.8)	1327 (49.1)	289 (38.4)	
1	1321 (38.3)	1025 (38.0)	296 (39.4)	
2	359 (10.4)	256 (9.5)	103 (13.7)	
≥3	156 (4.5)	92 (3.4)	64 (8.5)	
Number of siblings when 2 years old (%)				0.0
0	1362 (39.5)	1130 (41.9)	232 (30.9)	
1	1498 (43.4)	1170 (43.3)	328 (43.6)	
2	415 (12.0)	294 (10.9)	121 (16.1)	
≥3	177 (5.1)	106 (3.9)	71 (9.4)	
Living situation (%)				0.0
Mother and father	2999 (86.9)	2365 (87.6)	634 (84.3)	
Mother	408 (11.8)	301 (11.1)	107 (14.2)	
Father	35 (1.0)	28 (1.0)	7 (0.9)	
Neither parent	10 (0.3)	6 (0.2)	4 (0.5)	
Clinic allocation (%)				0.0
SAU	599 (17.4)	475 (17.6)	124 (16.5)	
SUS	553 (16.0)	472 (17.5)	81 (10.8)	
SPA	509 (14.7)	397 (14.7)	112 (14.9)	
SWE	440 (12.7)	316 (11.7)	124 (16.5)	
SMU	362 (10.5)	328 (12.1)	34 (4.5)	
SNO	989 (28.7)	712 (26.4)	277 (36.8)	
Current citizenship of primary caretaker (%)				0.0
Switzerland	2260 (65.5)	1868 (69.2)	392 (52.1)	
Western	509 (14.7)	429 (15.9)	80 (10.6)	
South America, Africa, Asia	378 (11.0)	218 (8.1)	160 (21.3)	
Eastern Europe, Turkey, Russia	264 (7.6)	154 (5.7)	110 (14.6)	
Other	41 (1.2)	31 (1.1)	10 (1.3)	
Residence permit status of child (%)				0.5
Swiss resident	2668 (77.3)	2152 (79.7)	516 (68.6)	
Foreign resident	590 (17.1)	425 (15.7)	165 (21.9)	
Foreign resident weekly/yearly permit	146 (4.2)	95 (3.5)	51 (6.8)	
Foreign resident short stay	0 (0.0)	0 (0.0)	0 (0.0)	
Asylum seeker, temporary asylum granted	30 (0.9)	14 (0.5)	16 (2.1)	
Missing	18 (0.5)	14 (0.5)	4 (0.5)	
