# Supplementary material for: Evaluation of a caries prevention programme for preschool children in Switzerland: is the target group being reached?
Source: BMC Oral Health. 2021 Nov 30;21:609. doi: 10.1186/s12903-021-01969-3 (PMC8638191; doi:10.1186/s12903-021-01969-3)
Supplement: Supplementary file 4 — Additional file 4. Frequency distribution of dmft indices at the toddler and kindergarten check-ups. [file 12903_2021_1969_MOESM4_ESM.pdf]

Evaluation of a caries prevention programme for preschool children in Switzerland: Is the target group being reached?

Table 1: Number of children with specific numbers of decayed, missing or filled teeth (dmft-index) at the toddler check-up and at the kindergarten check-up. Frequencies are lower at toddler check-up because dmft is only available for children who attended the check-up. The number of children with dmft-index was 2356 at the toddler check-up and 3452 at the kindergarten check-up.

**dmft-value**	**No. at toddler check-up**	**No. at Kindergarten check-up**
0	2276	2700
1	15	220
2	20	124
3	4	89
4	20	68
5	5	38
6	4	50
7	6	41
8	2	26
9	0	26
10	1	21
11	0	12
12	2	15
13	1	7
14	0	7
15	0	1
16	0	4
17	0	1
18	0	2
